# Improving immunization in Afghanistan: results from a cross-sectional community-based survey to assess routine immunization coverage

**DOI:** 10.1186/s12889-017-4193-z

**Published:** 2017-04-04

**Authors:** Raveesha R. Mugali, Farooq Mansoor, Sardar Parwiz, Fazil Ahmad, Najibullah Safi, Ariel Higgins-Steele, Sherin Varkey

**Affiliations:** 1 United Nations Children’s Fund, UNOCA Compound, UNICEF Afghanistan Country Office, Jalalabad Road, Kabul, Afghanistan; 2 The Health Protection and Research Organization (HPRO), Kabul, Afghanistan; 3 Ministry of Public health, Kabul, Afghanistan

## Abstract

**Background:**

Despite progress in recent years, Afghanistan is lagging behind in realizing the full potential of immunization. The country is still endemic for polio transmission and measles outbreaks continue to occur. In spite of significant reductions over the past decade, the mortality rate of children under 5 years of age continues to remain high at 91 per 1000 live births.

**Methods:**

The study was a descriptive community-based cross sectional household survey. The survey aimed to estimate the levels of immunization coverage at national and province levels. Specific objectives are to: establish valid baseline information to monitor progress of the immunization program; identify reasons why children are not immunized; and make recommendations to enhance access and quality of immunization services in Afghanistan. The survey was carried out in all 34 provinces of the country, with a sample of 6125 mothers of children aged 12–23 months.

**Results:**

Nationally, 51% of children participating in the survey received all doses of each antigen irrespective of the recommended date of immunization or recommended interval between doses. About 31% of children were found to be partially vaccinated. Reasons for partial vaccination included: place to vaccinate child too far (23%), not aware of the need of vaccination (17%), no faith in vaccination (16%), mother was too busy (15%), and fear of side effects (11%).

**Conclusion:**

The innovative mechanism of contracting out delivery of primary health care services in Afghanistan, including immunization, to non-governmental organizations is showing some positive results in quickly increasing coverage of essential interventions, including routine immunization. Much ground still needs to be covered with proper planning and management of resources in order to improve the immunization coverage in Afghanistan and increase survival and health status of its children.

## Background

Immunization is one of the most cost effective public health interventions, saving millions of lives and averting illness and disability among children [1]. as a direct result of immunization, polio is on the verge of eradication and 31 of the 59 high risk countries for maternal and neonatal tetanus have eliminated the disease through tetanus toxoid vaccination [2]. For other diseases such as measles, deaths have drastically reduced; for example deaths from measles have reduced by 75% from 2000 to 2013 worldwide, meaning 15.6 million deaths were averted [3]. In addition to lowering child mortality, immunization programs have improved the primary care infrastructure in developing countries and empowered women to better plan their families, with consequent health, social and economic benefits [4].

But immunization is yet to realize its full potential, largely because success of an immunization program depends on high rates of acceptance and coverage [5]. By the end of 2014, 18.7 million children under the age of 1 year had not received three doses of diphtheria-tetanus-pertussis (DPT3), a combination vaccine against three infectious diseases in humans (diphtheria, pertussis or whooping cough, and tetanus) [6]. Three quarters of children who have not received DPT3 coverage are living in 15 countries, including Afghanistan. Global polio eradication efforts have made important headway and now polio is endemic in only two countries Afghanistan and Pakistan.

Despite progress in recent years, Afghanistan is lagging behind in realizing the full potential of immunization. In addition, maternal and neonatal tetanus continues to threaten the lives of mothers and children [7–9], polio is still endemic, and measles outbreaks continue especially among groups of internally displaces persons (IDPs). In spite of significant reductions over the past decade, the mortality rate of children under 5 years of age continues to remain high at 91 per 1000 live births [10].

It is important to consider the history and contextual challenges in delivering child vaccinations in Afghanistan. The immunization program in Afghanistan was launched in 1978 under the name of “Mass Immunization Program” through the Ministry of Public Health (MoPH) and was then gradually expanded with the aim of universal immunization coverage throughout the country. Conflict in the late 1970s had a negative impact on immunization and the program was further disrupted in 1999 under the Taliban regime, along with several other health services [11]. In 2001, the MoPH of the new interim authorities had the enormous challenge of building the health care system from scratch. The maternal mortality ratio was estimated at 1600 maternal deaths per 100,000 live births in 2002, at that time the highest in the world [12], the infant mortality rate and under five mortality rate were 96 and 137 per 1000 live births respectively [13]. Only 8% of infants received DPT3 vaccination in 2004 [14]. With an estimated population of 27.6 million [15] and an annual population growth rate of 2.0% [16], children under 5 years of age account for 20% of the population. In addition to being heavily dependent on external aid and having weak governance, the health sector also faces the challenges of armed conflict, natural disasters, and internal displacement of an estimated 1.2 million people [17]. Ongoing conflict continues to cause widespread disruption to health services [18].

While improvements in vaccination coverage are documented, there are large discrepancies between the reported administrative coverage, individual survey results, and WHO/UNICEF estimates. Administrative data indicates that the immunization coverage for all antigens in Afghanistan has been increasing since 2001 though is inconsistent with other estimates. For example, DPT3 was estimated at 48% coverage in 2002 and increased to 101% in 2013. Frequent outbreaks of measles during the past two to 3 years also put the administrative coverage under question. While the administrative data shows high rates of coverage, the Multiple Indicator Cluster Survey (MICS) 2010 showed DPT3 coverage of 31% and full immunization coverage of 16% [19].

One of the important shortcomings for calculating immunization coverage in Afghanistan is the absence of accurate population data and therefore the number of target children. The last census held in the country was in 1979. For the last 35 years, the Central Statistics Organization is using projected figures and there is a high degree of uncertainty for figures available. Coverage levels for immunization are derived from administrative data and population estimates; wide-ranging population estimates present an enormous challenge for planning the immunization program in Afghanistan. Therefore, the MoPH, with support of UNICEF, developed and conducted a nationwide coverage evaluation survey to obtain reliable estimates of national and provincial level coverage of individual antigens and full immunization coverage. This paper presents the results of this survey along with an analysis of the challenges and solutions in scaling up immunization services in Afghanistan.

The survey aimed to estimate the levels of immunization coverage at national and sub-national (province) levels. The specific objectives of the survey are to: establish valid baseline information to enable the monitoring of progress of the immunization program; identify reasons why children are not immunized; and make recommendations to enhance the access and quality of immunization services in Afghanistan.

## Methods

### Materials and methods

The study was a descriptive cross-sectional household survey. The survey was carried out in all 34 provinces of the country between July and November 2013. Clusters for the survey were selected in both urban and rural settings as per the enumeration areas done by the Central Statistics Organization. The household survey had data on a number of households in each enumerated area, which was used to develop the sampling frame to select the clusters. Survey target groups and inclusion criteria included mothers of children aged 12–23 months. Mothers were interviewed at the time of survey to calculate the child immunization status for each of the provided antigens as well as the percentage of fully immunized children (either crude or valid coverage, overall and by the twelfth month of age). The vaccines included in Afghanistan’s routine immunization schedule and this survey include: Bacillus Calmette-Guérin (BCG) against tuberculosis; Pentavalent or five individual vaccines conjugated in one intended to protect against Haemophilus Influenza type B (bacteria causing meningitis, pneumonia and otitis), whooping cough (or pertussis), tetanus, hepatitis B and diphtheria; oral poliovirus vaccine (OPV); and the first dose of measles vaccine (Table 1 for vaccine schedule in Afghanistan). When a child failed to receive the next doses of the recommended antigens (three doses of Penta, OPV, and one dose of measles vaccine after taking BCG or Penta1), it is considered as a drop-out case.

**Table 1:** Childhood vaccination schedule in Afghanistan

SN	Age	Vaccine
1	Birth (0–11 Months)	BCG
2	Birth (as soon as possible within 14 days of life	OPV0
3	6 Weeks	Pentavalent 1, OPV1
4	10 Weeks	Pentavalent 2, OPV2
5	14 Weeks	Pentavalent 3, OPV3
6	9 Months	Measles, OPV4
7	18 Months	Measles

### Sample

The method was adapted from the WHO 30-cluster sampling and involved a two-stage sampling technique with a random selection of 30 clusters in each province based on probability proportional to size (PPS) and then a random selection of households in the selected clusters [20].

To estimate the sample size, the desired confidence interval was taken to be 95%. The level of precision of the estimates was set at ±10%. A design effect of 2 and an estimated routine coverage of (35) was assumed. The total sample size (minimum) was (175) using the standard formula for sample size calculation. The non-response rate of 20% was expected and hence, a final total of 210 children was required. In order to achieve this sample from 30 clusters, a total number of 7 children between 11 and 23 months of age were required. Thirty clusters were drawn in each province from the sampling frame using probability proportionate to size (PPS) method and accordingly, seven mothers/caregivers of children aged 12–23 were interviewed in each cluster. The 30 clusters provided 210 respondents in each group for each province. The national sample size was calculated to be 1020 clusters and 7140 mothers of children aged 12–23 months in 34 provinces.

The expectation was to conduct 7140 interviews with caregivers of children aged 12–23 months at the time the survey, however, 16 (1.6%) clusters were not accessible due to active security threats or geographical constraints; for the remaining 7028, the interviewer identified a total of 6854 (97.5%) potential participants based on the annual event calendar (date of birth ascertained by the guardians of children aged 12–23 months) in 34 provinces. Of the 6854 caregivers of children identified, further review revealed 206 children were older than 23 months and 502 children were younger than 12 months and therefore not eligible for the survey. In addition, 14 (0.2%) children were not eligible because the caregiver did not provide consent, 6 partially responded to the questionnaire with no data on immunization and the responsible caregiver for one child was not available for interview after being identified. Finally, 6125 (87.1%) children aged 12–23 months at the time of the survey were identified as eligible and interviews were conducted with the caregiver.

### Data collection and analysis

WHO Survey questionnaires for expanded program on immunization (EPI) coverage was adapted to include the national immunization schedule and questions on socio-economic status of household, and pilot tested before use [21]. The questionnaires included questions about demographic information of the respondent and of the index of the child followed by information about immunization. There were separate questions for collecting data on immunization from the immunization cards and from the mothers’ recall; questions on socio-economic status of the responding mother or caregiver were also added to the standard questionnaire. Both questionnaires were translated to Pashto and Dari languages. Questionnaires were field tested. Standard weighting method was used to derive the national estimates. In analysis of data of this survey, the principle component analysis (PCA) method was applied to produce the wealth index the derived asset index from the PCA enabled grouping of households into quintiles, to reflect different levels of socio-economic status. Key immunization results were cross tabulated with wealth quintile and presented in tables and graphs for child immunization data.

### Determinants of vaccination coverage at provincial level

In order to determine the association of the possible factors with variation of the immunization coverage at provincial level, bivariate as well as linear regression analysis was done. As outcome variables, Penta-3 coverage was used. As potential explanatory variables, a number of available indicators were explored such as the proportions of the urban versus rural population, education of the mothers/caregivers.

### Ethics

The ethical clearance was obtained by Institutional Review Board, Afghanistan National Institute of Public Health (ANIPH), Ministry of Public Health. This EPI coverage survey was designed and planned by the General Directorate of Preventive Medicine and the National EPI Management team of the Ministry of Public Health of the Islamic Republic of Afghanistan. The survey followed globally accepted standards promoted by WHO for EPI survey design, data collection, and reporting. Funding and technical support was provided by UNICEF.

## Results

### Background characteristics of participant mothers and children

The distribution of the sampled population was 15% urban and 85% rural. Nationally, 85% of mothers have no education, 3% attended some schooling but with no mention of years, 6% of respondents report they attended primary school 5% secondary school and 1% of mothers have above high school education (Table 2). Education level differs by locality; 12% of women in rural areas and 30% in urban areas have attended some schooling. This difference is statistically significant (*P* < 0·001). There are disparities at provincial level in mothers’ education with Kunar (0.5%), Badghis (1%), Urozgan (1.1%), Ghor (1.0%) and Paktika (3.5%) having the lowest proportion of mothers who attended some years of school, while Daikundi and Nimroz (32.7% each), Kabul (29.8%), Herat (24.6%) and Kapisa (22.4%) had the highest proportion of mothers who attended some schooling.

**Table 2:** Background characteristics of surveyed mothers of and children aged 12–23 months

	Number	Percent
Location		
Urban	898	15%
Rural	5227	85%
Total	6125	100%
Sex of child		
Female	2642	43%
Male`	3462`	57%
Missing data	21	0%
Total	6125	100%
Education of mothers		
None	5202	85%
Some school (years unknown)	170	3%
Primary	360	6%
Secondary	331	5%
Higher	62	1%
Total	6125	100%

### Vaccination card possession and retention rate

Two-thirds (66%) of mothers presented cards for their children to the surveyors, another 14% report they did have a vaccination card but could not present at the time of the survey. Therefore a total of 80% children had ever possessed a vaccination card for the index child. The proportion of mothers able to present the card to the interviewers varied from province to province; among the provinces Paktia with 99%, Panjsher at 97%, and Balkh and Samangan at 96%, had the highest rate of mothers presenting the vaccination card while Nooristan (14%), Helmand (17%), Urozgan (23%), Ghor (39%), and Badghis and Kandahar with 47%, had the lowest rates.

### Coverage by antigen

Firstly, crude coverage was calculated which includes all doses of each antigen received by the child irrespective of EPI-recommended age and/or interval. It also included immunization doses recorded on the card or confirmed through history given by the mother. Nationally, 51% of children participating in the survey received all doses of each antigen irrespective of the recommended date of immunization or recommended interval between doses. The coverage rates also show variation between provinces with Farah (2.5%), Nooristan (7.5%), Helmand (7.8%), Urozgan (15.3%), Ghor (19.4%), Zabul (27.4%), and Kandahar (37.4%) showing the lowest fully immunization coverage while Paktia (86.8%), Panjsher (85.7%), and Kapisa (82.7%) had the highest coverage for all antigens. The crude coverage rates by specific antigen at the age of 23 months are 77.9% for BCG, 73.3% for Penta1, 59.7% for Penta3, and 58.8% for measles vaccine (Table 3).

**Table 3:** Factors associated with immunization status of children aged 12–23 months

Crude Fully Immunized (card + history *n* = 6125)							
		Yes		No		Total	*P* value
Locality		%		%		%	
Urban		61.8		38.2		100	<0.001
Rural		49.0		51.0		100	
Total		51		49		100	
Education of mother							
Yes		65.9		34.1		100	<0.001
No		48.7		51.3		100	
Total		51		49		100	
Wealth quintile	Richest	2	3	4	Poorest	Total	
No	40.0	43.8	46.5	53.0	62.0	49.0	<0.001
Yes	60.0	56.2	53.5	47.0	38.0	51.0	
Total	100	100	100	100	100	100	
Not Immunized (*n* = 6125)							
Locality		Yes		No		Total	*P* value
Urban		9.6		90.4		100	
Rural		19.5		80.5		100	
Total		18.3		81.6		100	
Education of mother							
Yes		4.2		95.8		100	<0.001
No		20.1		79.9		100	
Total		18.3		81.6		100	
Wealth quintile	Richest	2	3	4	Poorest	Total	
No	87.9	85.6	84.9	81.9	66.3	81.6	<0.001
Yes	12.1	14.4	15.1	18.1	33.7	18.3	
Total	100	100	100	100	100	100	

The Fully Immunized Coverage (FIC) – as defined by WHO as BCG, Penta, OPV 1 2 3, and Measles [22] – was also calculated. Nationally, the FIC by the age 1 year is 36.3%. Overall, it seems girls are almost equally served by the vaccination program; there is about 2 percentage point difference in the coverage of BCG vaccination between males (77.2%) and females (79.1%).

### Incomplete vaccination coverage and dropout rates

About 30.7% of children were found to be partially vaccinated at the time of survey. The dropout rate is 23.4% for BCG-Penta3, 18.6% for Penta1-Penta3 and 24.5% for BCG-measles. Penta1-Penta3 drop-out rate ranges for some provinces; for example, Farah (50%), Nooristan (43%), Urozgan (38%), Zabul (36%), Faryab (31%), Daikundi (25%), Helmand (24%), Sar-e-Pul (23%), and Logar (20%) is higher than the national average of 13%.

### Left-out rate

The rate of no/zero immunization among children aged 12–23 months of age at national level is 18.3%. There is also considerable variation in provinces in the proportion of children who had not received any vaccination at all. Nooristan, Helmand, Urozgan, Zabul, Ghor, Badghis, Farah, and Kandahar provinces have the highest rate of children who never received a vaccine, while Nimroz, Paktia, Balkh, Panjsher, Bamyan, Jawzjan, Herat, Laghman, and Kapisa have the lowest rate of children with zero immunization.

### Reasons for left-outs and drop-outs

**Fig. 1:**
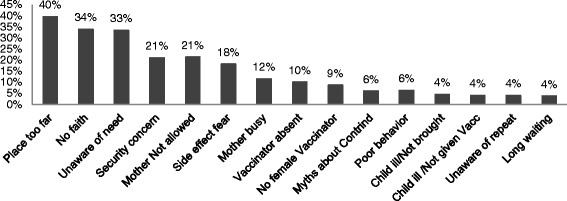
Reasons for no immunization among children 12–23 months

The survey reveals that 18.3% of children never received any vaccine. Mothers of children who never received vaccines were asked about reasons for not getting the child immunized. The major reasons given by mothers for never vaccinating their children included: place for vaccination being too far (40%), no faith in immunization (34%), unaware of the need for vaccination (33%), concerns about conflict-related security (21%), and not being allowed to go to a clinic without a male family member or *mahrahm* (21%). Other reasons mentioned were fear of side effects (18%), being too busy to take the child for vaccination (12%), vaccinator was absent (10%), and/or absence of female vaccinator (9%) at the health facility (Figure 1). Immunization services are free in Afghanistan and cost was not mentioned as an issue.

Nooristan, Kunar, Badghis, Farah, Sari-Pul, Urozgan Kabul, Kapisa, and Nangarhar are provinces where “place was too far” was mentioned most as a reason for no immunization. No faith in immunization or lack of awareness of the need to get the child immunized was mentioned most in provinces such as Helmand, Zabul, Laghman, Paktia, Parwan, Wardak, Badakhshan, Samangan, Takhar, Logar, and Panjsher. In Kandahar, Khost, Paktika, Ghor, Baghlan, and Faryab, the reason most mentioned for lack of immunization was the mother was either too busy or was not allowed to go to a health facility for vaccination for the child.

For the 30.7% of children partially vaccinated, reasons for partial vaccination included: 23% of the mothers reported the place to vaccinate child was too far, 17% said they were not aware of the need of vaccination, 16% said they had no faith in vaccination, 15% said the mother was too busy to take the child for vaccinations, and 11% reported they did so because of fear of side effects. Badakhshan, Nooristan, Bamyan, Daikundi, Kunar, Paktika, Farah, Kabul, and Khost are examples of provinces where “place was too far” was mentioned most as a reason for incomplete immunization. No awareness or no faith in immunization of the need to get the child immunized was mentioned most in provinces such as Helmand, Badghis, Sari-Pul, Zabul, Laghman, Urozgan, Samangan, Takhar, and Nimroz. In Baghlan, Ghazni, Ghor, Kapisa, Nangarhar, and Paktia the reason most mentioned for incomplete immunization was the mother was too busy to take the child for follow up vaccination doses.

Immunization status has a statistically significant association with a number of factors for children aged 12–23 months. Immunization coverage is higher among children living in urban areas (61.8%) compared with children living in rural areas (49.0%) for crude coverage by the age of 23 months, with a difference that is statistically significant (*p* < 0.001). Similarly, lack of immunization is associated with living in rural areas (*p* < 0.05). Immunization coverage or lack of it is positively and negatively associated with education of mothers or lack of education respectively. Crude coverage is 65.8% for children whose mothers attended some schooling while for those whose mothers did not attend any schooling is 48.7% (*p* < 0.001). Zero immunization is higher among children whose mothers did not obtain any education compared with those who had some education (20.1% vs 4.2%), also statistically significant. Wealth and socio-economic status are associated with immunization status: crude fully immunization coverage is about 22 percentage points higher for the wealthiest quintile (60%) compared to the poorest quintile (38%) and is statistically significant (*p* < 0.001). Lack of immunization is significantly different (*p* value <0.001) by wealth quintile, with 33.7% of the children with zero vaccination belonging to the poorest quintile while only 12.1% of the children in the wealthiest quintile have zero immunization.

## Discussion

The innovative mechanism of contracting out delivery of primary health care services in Afghanistan, including immunization, to non-governmental organizations is showing some positive results in quickly increasing coverage of essential interventions, including routine immunization [23].

The vaccination card is an inexpensive yet effective tool for systematically recording the vaccines received by a child. It is very helpful for the vaccinators to determine the timing and kind of vaccines to be given and can serve as a source of information as well as a reminder for caregivers. This survey found the card retention rate was 66% in Afghanistan and varied between provinces. A study from Pakistan had only 33% of retention rate, the important determinant of this low rate was bigger size of the household [24]. A similar study from Uganda showed 66% retention rate, and also confirmed that mother’s delivery in the health facility and former usage of health services for antenatal care were factors related to improved retention rate of the card [25]. One of the risks of low retention is early immunization; lost card was the major cause of early immunization, from a study of Bangladesh [26]. The same study showed 84% of retention rate of the card with DPT3 coverage of 75%. The design, form, and content in the card also have an effect on retention rate [27, 28]. Results from this survey highlight the need to focus on improving the distribution of cards to ensure women receive them for their children, especially women giving birth outside of health facilities, approximately half of births in Afghanistan [29]. Retention of the vaccination card is also critical in Afghanistan, which can be further promoted by health workers and community health workers.

FIC before the age of one is an important measure of quality of the program as it is a measure of children receiving timely protection from vaccine preventable diseases [30]. With card only, the FIC before the age of 1 year was 36% only, which means that only 1 in 3 children receive timely protection with vaccination. These findings call for greater attention from immunization stakeholders on issues such as building capacity of immunization providers to aim for FIC in their catchment areas through improved national and sub-national planning, follow-up, and monitoring. It also requires demand-side interventions to ensure that caregivers understand the value of vaccination and seek immunization services in a timely manner [30].

**Fig. 2:**
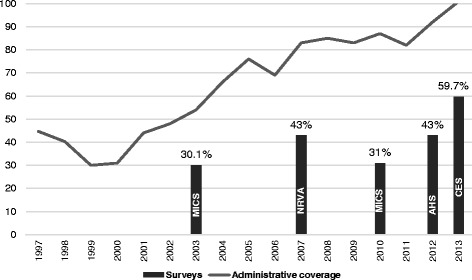
Routine immunization coverage for Afghanistan according to recent surveys and administrative data

Though the administrative reported coverage on routine immunization in Afghanistan indicates a progressive improvement, various independent surveys such as National Risk Vulnerability Assessment (NRVA), Multi-Indicator Cluster Survey (MICS), Afghanistan Health Survey (AHS) and Immunization Coverage Evaluation Survey (CES) show very low immunization coverage at the national level. The reported administrative coverage by the MoPH in 2012 is almost 85% for Penta3 coverage among children less than 2 years, substantially higher than the AHS survey reporting only a 43% coverage for Penta3. The present survey shows a slightly better trend for childhood immunization compared to the other surveys conducted in the previous years (Figure 2).

This rate of unvaccinated children (one in five children) shows that in spite of improvements in access as evident from the large proportion of the children having received BCG and Penta1 vaccines, still a large portion of children have no access to any vaccination. The set target for childhood immunization coverage is 90% for all antigens at the national level. These findings highlight the need for immunization partners to step up efforts in reaching out to these children. In the Afghan context, there is need for innovative approaches to reach communities who are uncovered by the health services due to security and tough geographic terrain [31]. This can be achieved through greater coverage in hard-to-reach areas by increasing outreach sessions and covering uncovered areas.

The dropout rate of 23.4% between BCG-Penta3, 18.6% from Penta1- Penta3 is very high, inferring a huge missed opportunity where children who are initiated with immunization do not receive subsequent doses in order to achieve the necessary levels of protective immunity. About 10 of 34 provinces (29%) have a high Penta3 drop-out rate and 16 of 34 provinces (47%) have a high drop-out rate for measles. There is an urgent need to institute mechanisms to track these children who had initially utilized the services and improving the regularity and predictability of immunization sessions along quality improvements in order to reduce the drop outs [32, 33].

Many studies confirm that the immunization coverage is associated with many economic and demographic factors such as parental education, area of residence, and sex of children, ethnicity and wealth of households. Family wealth status has been shown repeatedly to influence the chances of a child being immunized in many developing countries. Children from the poorest families have a significantly less chance of being vaccinated with the possible underlying cause being that the poorest are disadvantaged by the logistics and time required as well as the opportunity costs involved in accessing the immunization services [34, 35]. Data from Afghanistan also reveals similar inequities in immunization. While 60% of children from the richest quintile have full immunization coverage, only 38% of children from the poorest quintile have access. Afghanistan has lowest female literacy levels in the world; as per the Education Interim Plan 2011–2013 of the Ministry of Education, the adult female literacy is only 15% [36, 37]. Results from this survey also match this finding. It is important to take into account the context and history of Afghanistan with regards to female education; while public education has been rolled out for both boys and girls across the country since 2002, opportunities for female education prior were much scarcer, meaning many women in child-bearing years had little access to education.

Poor access and distance from vaccination services was the most frequently cited reason for not accessing immunization services. This reason is followed by communication and information reasons. Very specific to the context of Afghanistan is that women may not be allowed to go out alone. These reasons for not accessing immunization point to a need to improve both immunization system coverage and proximity to communities as well as to strengthen multi-pronged communication efforts [38].

A Cochrane review suggests that interventions oriented to community and patient, provider oriented and health system interventions are all needed to improve the coverage levels [39, 40]. A comprehensive approach including both demand as well as supply side interventions are required in Afghanistan to further accelerate immunization coverage and sustain past gains.

### Limitations

Exclusion of insecure and inaccessible clusters from the sampling frame where access to services is already compromised may have biased the results upward. Other potential for measurement bias were addressed through training of surveyors and close supervision of the data collectors during data collection. The fact that one third of children did not have immunization card and the interviewers relied on the recall of mothers, has the potential for information bias; to address this calendar of local annual events such as time of Eid festival was used to help mothers correctly time the child age and his/her immunization schedule.

More boys participated in this survey than girls, the reason behind which is was not explored, may have affected the results upward. The provincial comparison of the immunization services is done by considering the crude coverage of the Immunization Evaluation Survey 2013 irrespective of the immunization schedule. However, if one takes into account the other aspects of vaccine administration such as valid dose (provision of vaccine respective of the immunization schedule and children’s age), the actual immunization coverage will further decline.

## Conclusion

The survey findings are informative to direct efforts to accelerate the coverage of immunization in Afghanistan and further consolidate gains in the past decade. With ongoing polio transmission, measles outbreaks, and high child mortality, the country needs to prioritize both immediate plans to cover more children with all the vaccines, and a long-term plan of building immunization health systems. Immediate plans should focus on equity, with special attention to low coverage provinces for periodic intensified routine immunization campaigns and ways to expand outreach and mobile vaccination sessions to uncovered/poorly covered areas. Long term interventions must aim for robust cold chain vaccine logistics management system, permanent capacity building infrastructure, and human resources in terms of female vaccinators.

Communication is a paramount, cross-cutting strategy which needs to incrementally enhance knowledge through a multi-pronged approach. Findings from this survey suggest that the following areas must be prioritized:

- Given the complex factors affecting routine immunization, focused research and studies are needed to understand perceptions and feasible approaches to communicate with diverse groups.
- Social mobilization must be further leveraged to increase demand, learning from other initiatives such as polio eradication efforts.
- Demand promotion should have a special focus on building g confidence among caregivers in the quality and reliability of the services provided, and ensure that they have the necessary information and motivation to complete the immunization schedule.
- Demand promotion must also include capacity development and in-service performance support to improve the interpersonal communication and community engagement skills of frontline workforce (e.g., community health workers) and health service providers. Reminders for caregivers have been demonstrated to increase uptake and reduce loss to follow up in several low- and middle-income contexts.

Continued ownership at the Ministry level is needed to ensure the accountability of implementing NGOs including at the provincial and district levels. Focus of this area to include concurrent monitoring, periodic reviews and annual performance review by grant contracting management unit of the Ministry will facilitate coverage from life-saving VPDs. In addition, continued technical and financial support from donors and partners is vital as Afghanistan works to strengthen its routine immunization system.

Much ground still needs to be covered with evidence-based planning and management of resources in order to improve the immunization coverage in Afghanistan and improve survival and health status of its children.
